# Evaluating movement-based methods for estimating the frequency and timing of parturition in mule deer

**DOI:** 10.1186/s40462-024-00450-4

**Published:** 2024-01-19

**Authors:** Tabitha A. Hughes, Randy T. Larsen, Kent R. Hersey, Madelon van de Kerk, Brock R. McMillan

**Affiliations:** 1 Department of Plant and Wildlife Sciences, Brigham Young University, 4105 Life Sciences Building, Provo, UT 84602 USA; 2 Utah Division of Wildlife Resources, 1594 W North Temple, Suite 2110, Salt Lake City, UT 84116 USA; 3 School of Environment and Sustainability, Western Colorado University, Kelley Hall 144, Gunnison, CO 81231 USA

**Keywords:** Mule deer, Parturition timing, Movement, *Odocoileus hemionus*, Global positioning system, Utah, Pregnancy rate, Ungulate

## Abstract

**Background:**

Information on reproduction of harvested species such as mule deer (*Odocoileus hemionus*) is vital for conservation and management. Furthermore, parturition in ungulates may be detected using patterns of movement logged by GPS transmitters. Several movement-based methods have been developed to detect parturition in ungulates including the Peterson method, behavioral change point analysis (BCPA), rolling minimum convex polygons (rMCP), individual-based method (IBM), and population-based method (PBM). Our objectives were to (1) test the accuracy and the precision of each previously described method and (2) develop an improved method optimized for mule deer that incorporated aspects of the other methods.

**Methods:**

We determined parturition timing and status for female mule deer fitted with GPS collars and implanted with vaginal implant transmitters (VITs). We used movement patterns before and after parturition to set movement thresholds for each movement-based method. Following model training, we used location and birth date data from an external dataset to test the effectiveness of each movement-based method. Additionally, we developed a novel method for detecting parturition called the analysis of parturition indicators (API). We used two regression analyses to determine the accuracy and precision of estimates generated by each method.

**Results:**

The six methods we employed varied in accuracy, with the API, rMCP, and BCPA being most accurate. Precision also varied among methods, with the API, rMCP, and PBM generating the most precise estimates of parturition dates. The API and the rMCP performed similarly and better overall than any of the other existing methods.

**Conclusions:**

We found that movement-based methods could be used to accurately and precisely detect parturition in mule deer. Further, we determined that the API and rMCP methods had the greatest overall success at detecting parturition in mule deer. The relative success of the API and rMCP may be attributed to the fact that both methods use home range size to detect parturition and are validated using known parturition dates of collared deer. We present the API as an efficient method of estimating birth status and timing of parturition of mule deer fitted with GPS transmitters, as well as affirm the effectiveness of a previously developed method, rMCP.

**Supplementary Information:**

The online version contains supplementary material available at 10.1186/s40462-024-00450-4.

## Background

Reproduction is a critical life-history event for all organisms. Accurate estimates of successful reproduction, including when and where animals are giving birth can provide insights into the ecology of wildlife species [1, 2]. In large herbivores, the recruitment of young is highly variable relative to adult survival, and thus can have a large impact on population dynamics [3, 4]. As climatic variability increases so does the synchrony of births in ungulate populations; offspring must be born late enough to avoid harsh winter conditions, but early enough to allow sufficient time for young to grow before the following winter [5, 6]. Therefore, parturition is often highly synchronous for ungulates in temperate climates [7]. The synchronous nature of parturition may also serve to “swamp” predators and reduce overall neonatal mortality [6, 8]. An understanding of which females from a population give birth each year as well as the timing of parturition can therefore provide important insights into neonate survival and changes in population dynamics.

Frequency and timing of parturition in ungulates can be determined in a variety of ways. Blood samples collected from study animals, for example, may be used to determine pregnancy status [9]. Likewise, ultrasonography may be used to determine pregnancy status and even litter size in ungulates [10, 11]. One way of determining timing of parturition is by observing young at heel, either from the ground or during aerial surveys and estimating age of those young animals [7, 8, 12, 13]. Timing can also be determined by opportunistically catching neonates and using behavior and morphological characteristics (e.g., hoof growth, coordination, presence of a placenta, etc.) to determine age and approximate date of birth [14–16]. Additionally, the use of vaginal implant transmitters (VITs) can yield exact times and dates of parturition by detecting changes in light and temperature once expelled during a birth event [17–21]. While these methods may be effective at estimating timing of parturition, they are expensive, time-intensive, and highly invasive in some instances.

As technology improves, location data from animals with GPS collars are being acquired at increasingly high fix rates, offering an opportunity to estimate parturition based on changes in movement patterns of parturient individuals. Following parturition, female ungulates typically reduce their rate of movement and occupy a smaller range for up to seven days depending on the species [22, 23]. Several statistical methods have been developed to detect the change in movement associated with parturition and infer the timing of parturition from GPS data [24–27]. Understanding the relative accuracy of these methods may be useful for optimizing the ability to detect parturition, while minimizing the money and effort required.

Methods often detect parturition in a variety of species of ungulates by identifying breakpoints in different movement parameters. These methods include the Peterson method, behavioral change point analysis (BCPA), rolling minimum convex polygons (rMCP), the individual-based method (IBM), and the population-based method (PBM; Table 1). The Peterson method draws upon previous evidence supporting movement rate as a viable metric for identifying parturition and introduced two rules for estimating dates of parturition for mule deer (*Odocoileus hemionus*) [24, 26]. The first rule identifies the date of parturition when velocity is reduced > 46% and persists for $\ge$ 3 days, while the second rule uses a total daily movement threshold of < 700 m that persists for $\ge$ 3 days to identify parturition. [26]. Both rules were validated using parturition data from deer implanted with VITs, and rule one was found to be 89% accurate within four days while rule two was 77% accurate [26]. The BCPA is a statistical package that incorporates both velocity and turning angle into a measure of persistence velocity to identify significant changes in animal movements [28]. It was later modified to detect parturition in moose (*Alces alces*) by setting a moving window of 50 datapoints, the sensitivity parameter to 0.3 (0.5 if no changepoints were identified), and the cluster width to 48 h. Using aerial surveys, the BCPA was 99% accurate in estimating parturition for moose [27]. The rMCP approach uses minimum convex polygons from GPS locations over rolling 24-h windows to detect parturition, and was also confirmed using aerial surveys of collared moose to be 99% accurate [27]. The IBM is a method adapted from theBCPA that was developed for woodland caribou (*Rangifer tarandus*),was validated by aerial surveys to be 97% accurate in the initial study, and was later found to be 77% accurate for a related species, barren-ground caribou, in a subsequent study [25, 29]. The IBM detectss parturition by fitting models of step lengths of individuals to one of three a priori models, wherein the first model represents a female who gave birth, the second represents a female who did not give birth, and the third represents a female who gave birth but lost the young shortly after [25]. The PBM was also developed for woodland caribou and uses movement rate thresholds calculated by a subset of females with known parturition dates to estimate parturition for other animals in the population. The PBM identifies breakpoints by smoothing movement rate across a rolling 3-day window, and was validated via aerial surveys to be 100% accurate for woodland caribou in the initial study and 81% accurate for barren ground caribou in a subsequent study [25, 29].

**Table 1:** Five movement-based methods used to estimate parturition in ungulates

Method	Species	Parameters	Citations
Peterson	Mule deer (*Odocoileus hemionus*)	Velocity	Peterson et al., [26]
Behavioral change point analysis	Moose (*Alces alces*)	Velocity + turning angle	Nicholson et al., [27]
Rolling minimum convex polygons	Moose (*Alces alces*)	Home range size	Nicholson et al., [27]
Individual-based method	Caribou (*Rangifer tarandus)*	Step-length	DeMars et al., [25]
Population-based method	Caribou (*Rangifer tarandus)*	Step-length	DeMars et al., [25]

Methods are listed with the species of ungulate that they were developed or adapted for, movement parameters used, and references

The effectiveness of specific movement metrics and thresholds for detecting parturition likely varies by species. While many ungulates exhibit measurable changes in space use during parturition, species and regional specific differences in reproductive strategies may give rise to differences in female behavior following parturition. For example, directly after birth ungulate neonates will exhibit behavior along a continuum of “hiders” to "followers" [30]. Species such as caribou have follower offspring, in which young are extremely mobile and able to follow maternal females almost directly after being born; moose have offspring that are hiders for a short time after birth but switch to a follower strategy after leaving the birth site [31]; and mule deer have hider offspring, in which young stay relatively immobile and hide for the first few days of life while the mother spends most of her time away from them [30]. Due to these behavioral differences, methods developed for specific ungulates may not be as effective when transferring across species and some modification may be required[25, 32, 33]. Furthermore, methods using multiple movement metrics may be more effective than those using a single metric [34–36].

Although the timing of parturition of mule deer has previously been estimated using changes in daily movement rate and distance moved, the precision of these estimates are unclear. Mule deer, which occupy the western half of North America, are a species of both ecological and economic importance [37]. Nevertheless, mule deer populations have been in decline in recent years [38]. More effective and cost-efficient methods may be needed to understand the ecology of mule deer and inform management decisions regarding this species. In particular, improving the ability to understand the factors influencing neonate survival and recruitment of mule deer (such as timing of parturition and pregnancy rates) is of high consideration.

Although the Peterson method was developed for mule deer and is relatively accurate (89%), the use of multiple movement metrics instead of one may be more effective at estimating parturition. Additionally, the other four movement-based methods may be useful for detecting whether or not moose and caribou have given birth, but their applicability for mule deer are unknown. As such, the objective of this study was to improve or enable more accurate and precise estimates of the birth status and timing of parturition in mule deer using movement data. Specifically, we tested the five previously developed methods and a new method that we developed using known parturition dates of collared mule deer. We evaluated the performance of each method by determining both accuracy (i.e., the proportion of birth events correctly identified) and precision (i.e., the deviation of estimated dates of parturition from actual dates of birth). We hypothesized that movement-based methods developed specifically for mule deer would perform better than those developed for other species. Furthermore, we hypothesize that methods using multiple movement parameters to identify parturition events would perform better than relying on one. and therefore, this study would provide important recommendations to optimize the use of movement data to estimate both the occurrence and timing of parturition for mule deer.

### Methods

#### Study site

**Fig. 1:**
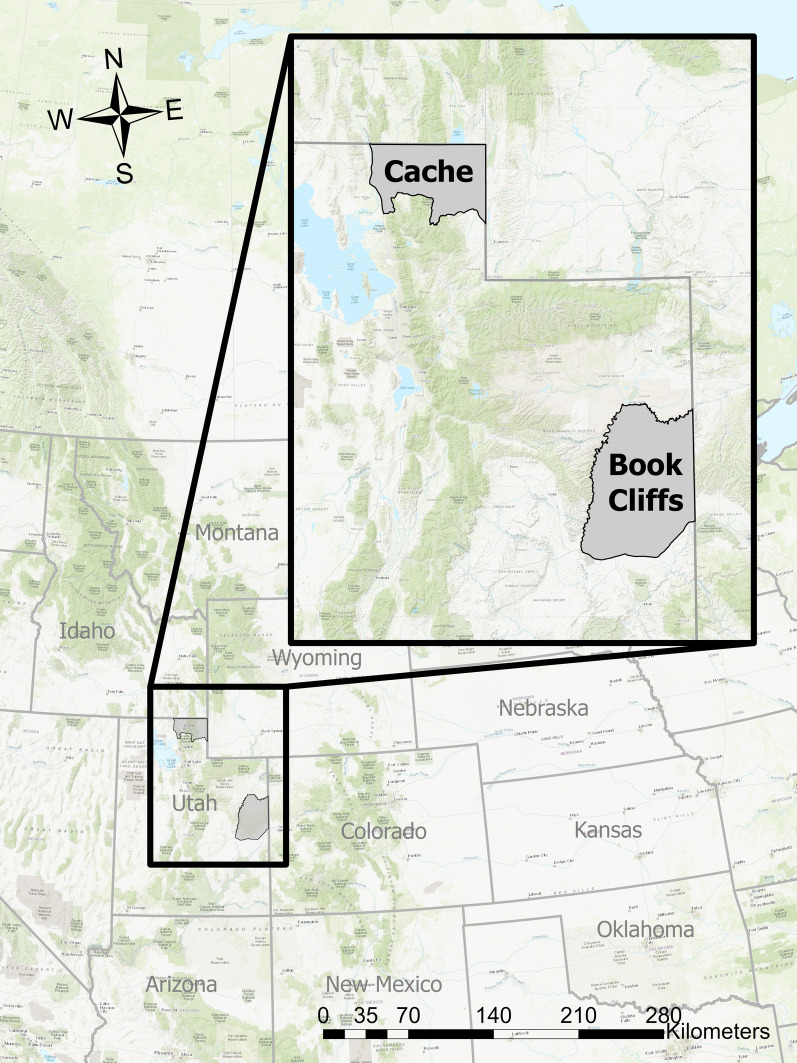
We captured, collared, and inserted vaginal implant transmitters into female mule deer (*Odocoileus hemionus*) on two study sites located in Utah, USA. Deer were captured during 2019–2021 in the Book Cliffs study site (located in west-central Utah), and during 2018–2020 in the Cache study site (located in northern Utah)

We captured female deer in two study areas located in Utah (Fig. 1). The first site, Cache, was located in northern Utah, between 42.0° and 41.4°N, and between − 111.0° and − 111.8° W. Average annual precipitation from 1900 to 2016 was 39 cm, and average temperatures ranged from -6° C in the winter to 17° C in the summer [39]. Elevation ranged from 1300 to 2800 m. The landscape in this area alternated from lower elevation hilly terrain consisting of bunchgrasses such as bluebunch wheatgrass (*Psuedoroegneria spicata)* and Idaho fescue (*Festica idahoensis*), and big sagebrush (*Artemisia tridentata*), to higher elevation forests consisting of mountain mahogany (*Cercocarpus ledifolius*), Douglas-fir (*Psuedotsuga menziesii*), Engelmann spruce (*Picea engelmannii*), and quaking aspen (*Populus tremuloides*) [40].

The second study area, Book Cliffs, was located in east-central Utah between 40° and 38.9° N, and between − 109.1° and − 109.7° W. Average annual precipitation from 1911 to 2016 in this area was 26 cm, and average temperatures ranged from − 5° C in the winter to 22° C in the summer [39]. Elevation ranged from 1400 to 3000 m. The landscape alternated between arid deserts and canyonlands consisting of mat saltbush (*Atriplex confertifolia*), bud sagebrush (*Artemisia spinescens*), galleta (*Hilaria ssp*.), and Indian ricegrass (*Achnytherum hymenoides*) to higher elevation escarpments consisting of pinyon pine (*Pinus edulis*), juniper (*Juniperus ssp.*), big sagebrush (*Artemisia tridentata*), Gambel’s oak (*Quercus gambellii*), mountain mahogany (*Cercocarpus ledifolius*), and Douglas-fir (*Psuedotsuga menziesii*) [40].

#### Capture

We captured adult deer during March of 2018–2020 in the Cache study area (*N* = 105) and during March of 2019–2021 in the Book Cliffs study area (*N* = 116). Individual adult females were captured using net gunning from a helicopter [41–43]. Following capture, deer were hobbled, blindfolded, and transported to a nearby processing station. We determined pregnancy status for each individual using a transabdominal ultrasound [10]. We inserted a vaginal implant transmitter (VIT) into each pregnant female and fitted all deer with a GPS tracking collar capable of communicating with the VIT (Model M3930U and Model G5-2DH, Advanced Telemetry Systems Inc., Isanti, MN, USA) [17]. Once collared, we immediately released deer from the site of processing. All animals were captured following standard Utah Division of Wildlife Resources protocols, guidelines from the American Society of Mammologists, and after review by the Institutional Animal Care and Use Committee at Brigham Young University [44].

#### Estimating parturition from VITs

During May–July following each March capture, we monitored collared females that survived to the birthing season (N = 191) for parturition events. After a VIT was expelled during parturition, a sensor detected the change in light and temperature and caused the female’s collar to trigger an email indicating that a neonate had been born. We waited at least four hours after notification before proceeding to the site of parturition to reduce the likelihood of neonate abandonment [45]. After arriving at the site of parturition, we first located the VIT, then systematically searched the surrounding area and the collared female’s most recent GPS locations for neonates or signs of neonates. Once we located one or more neonates, we estimated the parturition date and time for each of the collared females as the date and time the first “birth triggered” warning was indicated by the VIT. Occasionally, we would receive the birth notification earlier than expected (i.e., before the normal birthing season) or we would be unable to find neonates after locating the VIT, indicating that the VIT had most likely been expelled early. Because of the uncertainty surrounding the true date of parturition for deer who expelled the VIT prior to parturition, we excluded these females from our analyses.

#### Model training

We compiled GPS data from May 20 through July 31 for each female deer with a VIT from whom we successfully found neonates. Additionally, we included GPS data from any females that were not pregnant during March captures in order to test the ability for methods to identify barren (i.e., nonparturient) individuals. Each collar acquired a location approximately once every two hours, and locations were uploaded via satellite once every twelve hours. Individuals missing more than 12 fixes in a 48 h timeframe were excluded from our analysis (*N* = 2).

To test the effectiveness of different movement-based methods at detecting parturition in mule deer, we used a priori knowledge on mule deer movement patterns before and after parturition to alter the original parturition thresholds set by each method to better fit our dataset. In general, we found that mule deer exhibited decreased step-length, decreased velocity, increased turning angle, and used smaller home ranges immediately after parturition. Using information on these movement patterns as well as an initial exploration of the data using each method, we set movement thresholds for each method. For the PBM, we created a new movement rate threshold using GPS data from the three days following parturition for each collared doe in our study population Similar to the methods of Cameron et al., we bootstrapped the step-length threshold by generating a fawning threshold for a random selection of 10 individuals from our dataset, running this calculation 1000 times, and selecting the most common value generated [29].. We adjusted three parameters in the IBM to better fit our dataset, setting the minimum number of steps (min.adult) to 42, the maximum number of steps (max.adult) to 168, and the minimum steps before and after parturition (referred to as “int” parameter) to 24. After an initial exploration of the data using the BCPA method, we set the moving window to 60 locations (five days), the sensitivity parameter (K) to 0.3, the cluster width to 36 locations (three days), and selected Phi (Φ) as the representative movement metric using the bcpa [3, 46]. For the rMCP, we set the MCP threshold to 10 ha^2^, used a 48-h rolling window, and determined parturition when rolling MCPs remained at this size for 96 h (4 days). We kept the same movement thresholds of the Peterson method that were used in the original study as doing so was most effective for our dataset.

We further developed a method for detecting parturition in mule deer that will hereafter be referred to as the analysis of parturition indicators (API). In order to detect and estimate the timing of parturition, we developed parturition thresholds for three movement metrics (velocity, turning angle, and home range size). We used a paired two-sample *t*-test for means to determine differences in mean velocity, turning angle, and home range size for the three days following parturition compared to the time period prior to parturition (from the beginning of fawning season, May 20, to the date parturition occurred). We determined parturition thresholds to be the average velocity (km/hr), turning angle (radians), and home range size (95% MCPs) during the three days following parturition, plus the standard deviation for all study animals of which we were able to confirm the date of parturition. We rounded thresholds to the nearest 10 units (home range size), 0.01 unit (velocity), and 0.1 unit (turning angle).

#### Model validation

We used an external dataset consisting of known birthdates from 2023, which were collected from four additional populations of mule deer in Utah to test the effectiveness of the IBM, PBM, BCPA, rMCP, API, and Peterson methods [25–27]. The four populations used for model validation were located in different regions than the populations used to train models, with one population from central Utah (Nebo), one from east-central Utah (Nine Mile), and two from southeastern Utah (La Sal and San Juan). Parturition dates for collared deer in these populations were determined using the same methods that are described above for the training dataset (Knight et al. unpublished data, Brown et al., unpublished data). Fix rate success of this validation data was high, and 96.5% of all locations were collected precisely every 2 h. GPS data from collared deer with verified parturition dates (*N* = 98) was used to test the precision of the movement-based methods, while additional GPS data from collared deer who were confirmed to be not pregnant (*N* = 8) were included to test accuracy. We recognize that the low number of barren females relative to parturient females is a limitation of our data, however adult mule deer typically have very high rates of pregnancy regardless of environmental conditions [47].

We used modified movement thresholds and previously published procedures to estimate parturition events and parturition timing of female mule deer using the IBM, PBM, BCPA, rMCP, API, and Peterson methods [25–27]. Each analysis was conducted in R version 4.2.2 [48]. When testing the IBM and PBM, we used supplementary code provided in a later study to exclude models from the original study that detected neonate mortality as this was beyond the scope of our study [29]. For the IBM, PBM, and the Peterson method, we used the R package adehabitatLT to calculate movement rates and the distance traveled between subsequent GPS locations [49]. For the Peterson method, we chose to only test rule one as it was better performing in the initial study than rule two [26]. To test the API we calculated three-day rolling averages for velocity, and turning angle for each individual using the adhabitatLT package in R. Additionally, we calculated three-day rolling home range estimations (95% MCPs) for all study animals. The day of parturition was identified as the first datapoint in which average three-day rolling velocity was $<$ 0.05 km/hraverage turning angle was $>$ 1.8 radians, and when the three-day rolling home range size was < 30 hectares. If this pattern of locations was not present in the GPS data, then the individual was identified as not parturient. As with other movement-based methods, we used the API to estimate the timing and occurrence of parturition of collared mule deer with known parturition dates.

We used the lme4 package in R to determine the accuracy of each method using a mixed-effects logistic regression [50]. We modeled success at estimating parturition (validated with the VIT data) as a function of the method used with year and study area of individuals as random effects (intercept). We also determined the precision of each method using a second mixed-effects logistic regression, by modeling success at identifying the timing of parturition as a function of the method used and the days deviating from the true date of parturition (ranging from zero to seven days). Similar to the first model, year, study site, and individual were included as random effects (intercept).

## Results

We captured a total of 221 mule deer during March 2018–2021 and fitted each individual with a GPS collar. Six deer were not pregnant, while the remaining 215 deer were pregnant (97% pregnancy rate) and were each implanted with a VIT. Of the 215 pregnant mule deer, 30 died prior to parturition. We censored 75 individuals of whom we could not confirm timing of parturition, as a result of collar malfunctions, inability to access birth sites due to weather or remoteness of animal location, early expulsion or failure of VITs, or when we were unable to find neonates or evidence of neonates near the VIT expulsion site. We censored 18 deer who either produced stillborn offspring or had neonates that died within three days of parturition and censored 2 deer whose collars did not upload locations frequently enough to be included in further analysis. Location and time data from the remaining 90 deer were used to determine movement thresholds for the API, IBM, PBM, rMCP, BCPA, and Peterson methods. Location and time data from 106 additional deer (98 pregnant, 8 barren) from an external study were used to evaluate the accuracy of each method (i.e., whether or not parturition had occurred), the precision of estimated dates of parturition, and the movement thresholds at which parturition occurred.

Movement parameters (e.g., velocity, turning angle, and home range size) differed significantly before and after parturition. Prior to parturition, average velocity was 0.089 $\pm$ 0.009 km per hour (km/hr), while velocity after parturition averaged 0.033 $\pm$ 0.002 km/hr (*P* < 0.001). Average turning angle prior to parturition was 1.51 $\pm$ 0.01 radians, while turning angle after parturition was 1.90 $\pm$ 0.01 radians (*P* < 0.001). Finally, average three-day home range size prior to parturition was 79.6 $\pm$ 7.4 ha^2^, while home range size during the three days after parturition averaged 10.0 $\pm$ 1.9 ha^2^ (*P* < 0.001).

**Fig. 2:**
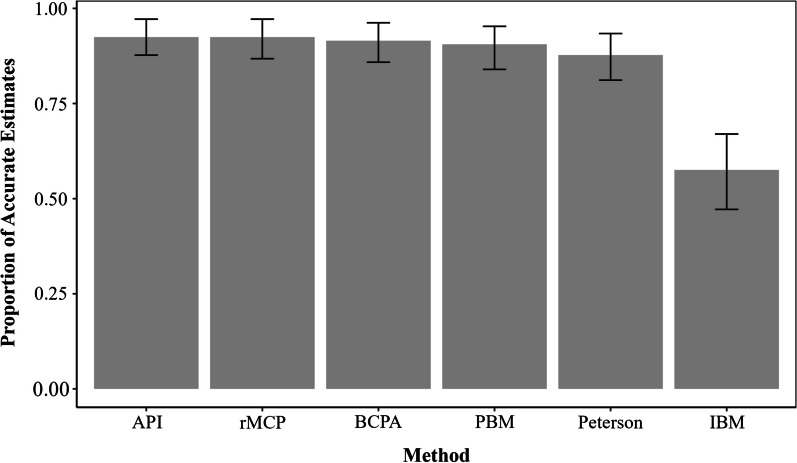
Accuracy of movement-based methods used to infer whether or not a female mule deer (*Odocoileus hemionus*) had given birth during 2023 in Utah, USA. Methods include the Peterson method, analysis of parturition indicators (API), rolling minimum convex polygons method (rMCP), population based method (PBM), behavioral change point analysis (BCPA), and the individual based method (IBM). The proportion of accurate estimates is the proportion of the study population whose status of parturition (i.e., parturient or barren) was correctly identified by each of the methods

There was some variation in accuracy among methods (*P* < 0.05; Fig. 2). The API (which was the intercept in each of our models) differed in accuracy from the IBM (*P* =  < 0.001), but did not differ from the Peterson method (*P* = 0.255), rMCP (*P* = 1.000), PBM (*P* = 0.623) or BCPA (*P* = 0.800). Methods also varied in their ability to correctly identify parturient females (sensitivity; Table 2) versus their ability to correctly identify non-parturient females (specificity; Table 2).Overall, the API had the best accuracy, followed in order of performance by the rMCP, BCPA, PBM, Peterson, and IBM.

**Table 2:** Performance of 6 movement-based methods for identifying parturition events and parturition timing for mule deer in Utah during summer of 2023

Method	Accuracy (%)	Precision (7 days)	Sensitivity (%)	Specificity (%)
Peterson	87.7 $\pm$ 3.2	78.4 $\pm$ 4.4%	89.8 $\pm$ 3.1	62.5 $\pm$ 17.1
BCPA	91.5 $\pm$ 2.7	34.7 $\pm$ 4.8%	99.0 $\pm$ 1.0	0.0 $\pm 0.0$
rMCP	91.5 $\pm$ 2.7	90.3 $\pm$ 3.1%	94.9 $\pm$ 2.2	50.0 $\pm$ 17.7
IBM	58.5 $\pm$ 4.8	70.7 $\pm$ 6.0%	59.2 $\pm$ 5.0	50.0 $\pm$ 17.7
PBM	90.6 $\pm$ 2.8	46.8 $\pm$ 5.1%	98.0 $\pm$ 1.4	0.0 $\pm$ 0.0
API	92.5 $\pm$ 2.6	89.2 $\pm$ 3.2%	94.9 $\pm$ 2.2	62.5 $\pm$ 17.1

Methods tested include the Peterson method, behavioral change point analysis (BCPA), rolling minimum convex polygons (rMCP), individual based method (IBM), population based method (PBM), and the analysis of parturition indicators (API). Accuracy is the percentage of female deer whose parturition status was correctly identified (*N* = 106), precision is the percentage of parturient deer whose date of parturition was correctly estimated within 7 days of the true date of parturition (*N* = 98), sensitivity is the percentage of parturient deer who were correctly identified as parturient (*N* = 98), and specificity is the percentage of barren deer who were correctly identified as non-parturient (*N* = 8)

**Fig. 3:**
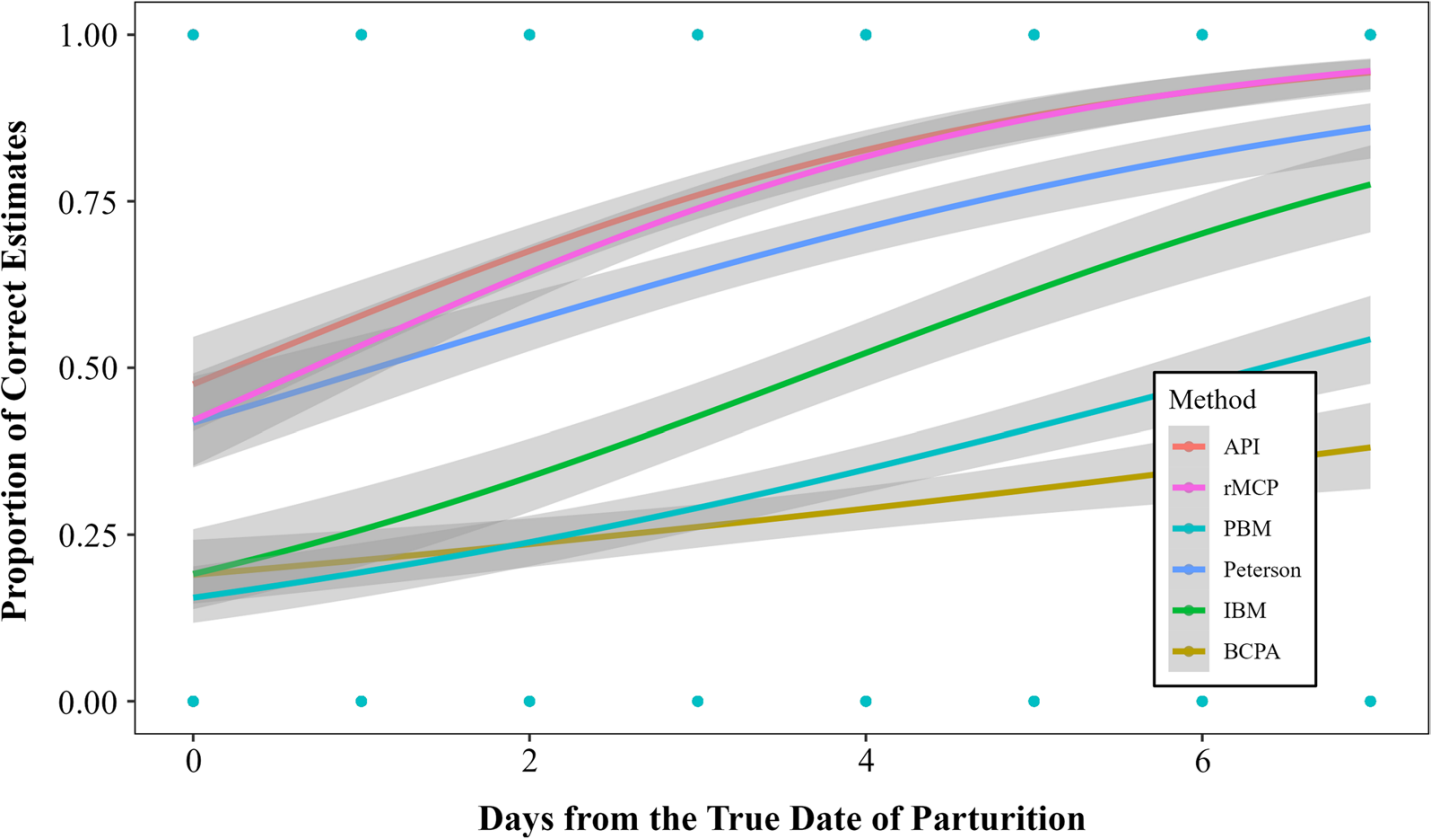
Proportion of correct estimates of the timing of parturition of female mule deer (*Odocoileus hemionus*) derived from various methods. Methods include the analysis of parturition indicators (API), behavioral point change analysis (BCPA), the individual based method (IBM), the Peterson method, the Population Based Method (PBM) and rolling minimum convex polygons method (rMCP). Precision changes as deviation from the actual date of parturition increases, and among methods (shaded areas represents the 95% confidence intervals). Timing of parturition for female deer was observed during 2023 in Utah, USA

**Fig. 4:**
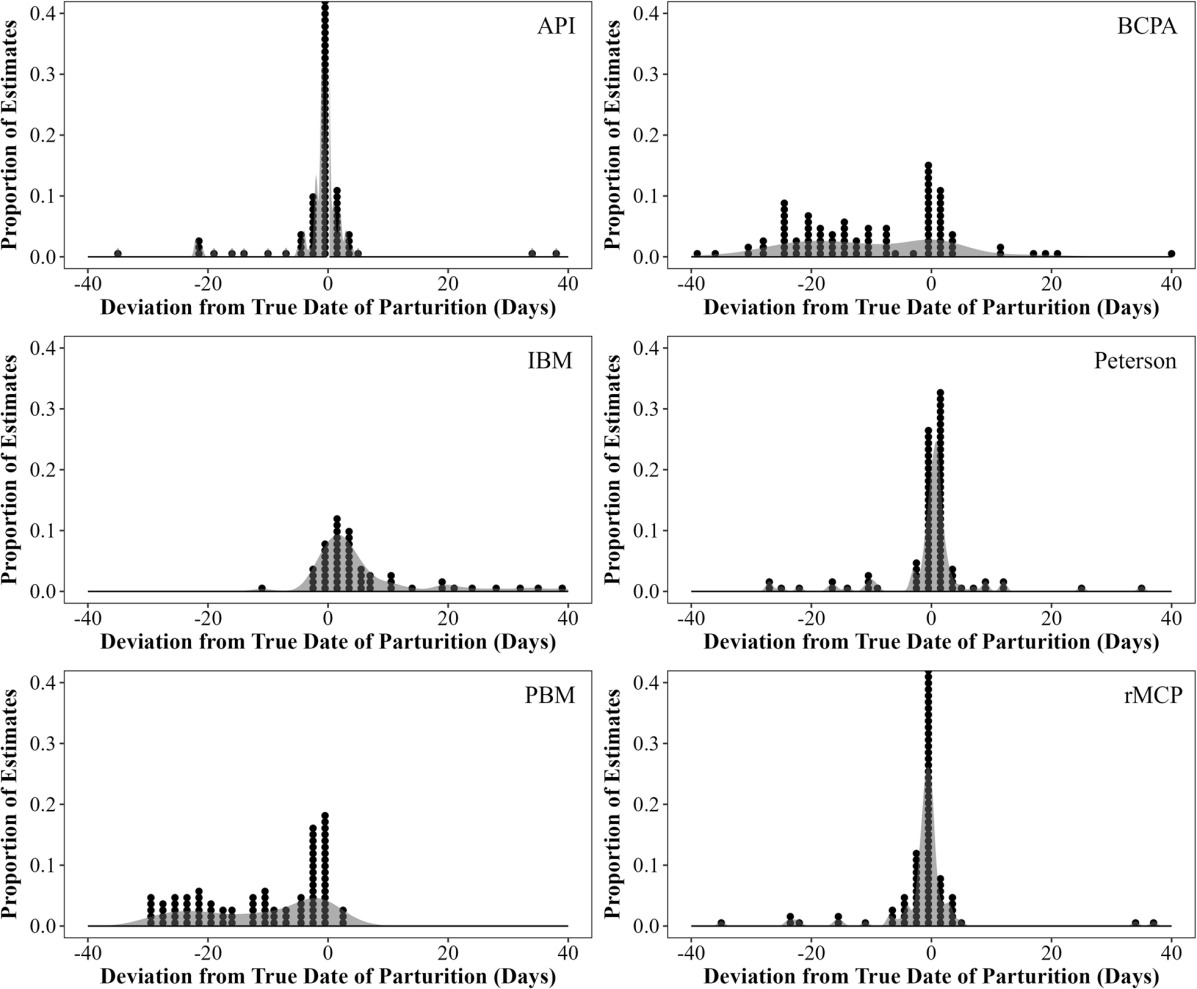
Deviation of estimated dates of parturition from the true dates of parturition for the analysis of parturition indicators (API), the behavioral change point analysis (BCPA), the individual based method (IBM), the Peterson method, the population based method (PBM), and the rolling minimum convex polygons method (rMCP). On average, dates calculated by the API, BCPA, IBM, Peterson, PBM, and rMCP were 3.6 (*CV* = 2.11), 14.6 (*CV* = 0.87), 9.6 (*CV* = 1.37), 5.3 (*CV* = 1.80), 12.1 (*CV* = 0.93) and 3.7 (*CV* = 2.03) days off from the true date of parturition, respectively. Estimated dates of parturition were derived using movement patterns of GPS collared mule deer (*Odocoileus hemionus*) with known parturition dates during 2023 in Utah, USA

**Fig. 5:**
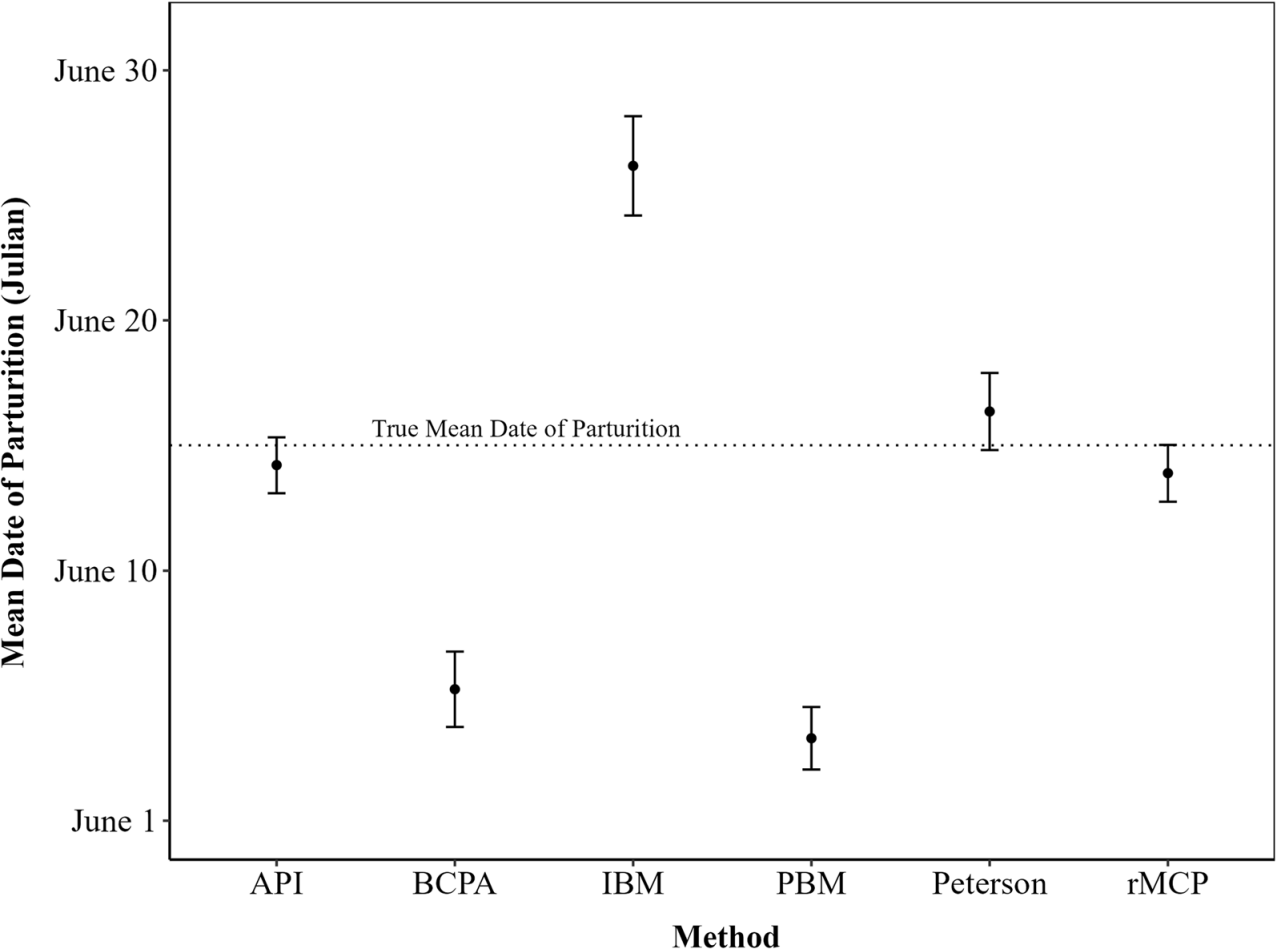
Estimates of mean dates of parturition for a study population of female mule deer (*Odocoileus hemionus*) during 2023 in Utah, USA. Dates of parturition are estimated using six different methods, the analysis of parturition indicators (API), the behavioral change point analysis (BCPA), the individual based method (IBM), the Peterson method, the population based method (PBM), and the rolling minimum convex polygons method (rMCP). The dotted line indicates the true mean date of parturition for the population, and error bars indicate the standard error of the mean for each estimate

Precision of estimated dates of parturition also varied between the API and other methods (*P* < 0.05; Fig. 3). The API and the rMCP had the highest precision of all methods, and the mean date of parturition estimated by these methods for the study population was less than one day from the actual mean date of parturition for the population (Fig. 4). Precision of the API differed from the BCPA (*P* < 0.001), the IBM (*P* < 0.001), the Peterson method (*P* < 0.001), but did not differ from the PBM (*P* = 0.117), or the rMCP (*P* = 0.251). In order of performance, the rMCP had the best precision, followed by the API, Peterson, IBM, PBM, and BCPA. The mean date of parturition for the study population in 2023 (the year data for model validation was collected) was June 15th, which was correctly estimated by the API, rMCP and Peterson method (Fig. 5). The BCPA estimated the mean date of parturition to be June 5th, the PBM estimated June 3rd, while the IBM estimated June 26th.

## Discussion

Using movement patterns to detect parturition allows for efficient estimates to be made of both the frequency and timing of parturition in ungulates. Although field methods and VITs may also provide accurate estimates of rates and timing of parturition, they can be time-intensive, expensive, invasive, and subject to failure [17, 51]. Therefore, an understanding of the relative accuracy and precision of movement–based methods of detecting parturition is necessary. To improve the detection of parturition using movement patterns, we determined the accuracy and precision of five previously developed methods that use differing movement metrics in estimating rates and timing of parturition using data from collared mule deer. We also developed and implemented a novel method for detecting parturition and estimating the timing of parturition in mule deer, called the analysis of parturition indicators (API). Consistent with our first hypothesis, there was considerable variation in both accuracy and precision among methods, with methods designed specifically for mule deer generally performing better than those designed for other species.

All six methods use differences in post-parturient behavior relative to pre-parturient movement patterns (i.e., normal movement patterns) to estimate whether parturition had occurred or not, however there was variation in success among the six methods we tested. The API and Peterson method had higher accuracy than the IBM but not other methods, while the API, rMCP, and PBM had the highest precision out of all methods. While the tendency to change behavior during and after parturition is common in ungulates, the variation in behavior among different species likely accounts for the variation in accuracy and precision we observed among methods [23, 52, 53]. The API and Peterson method were both developed specifically for detecting parturition in mule deer, while other methods used caribou or moose as the model species [24, 26]. While the Peterson method wasn’t the most effective method, it had higher precision than the BCPA, PBM, or IBM. Additionally, the specific parameters used to quantify the rapid decrease in movement following parturition may impact accuracy. The overall best performing methods, the API and rMCP, both incorporate home range size to detect parturition events, while the lower-performing methods (Peterson, IBM, PBM, and BCPA) used only movement-based metrics such as step-length, velocity and turning angle [25–27]. It appears that when estimating whether or not a mule deer has given birth, space use metrics may be more important than movement-based metrics.

The utility of including metrics such as home range size for estimating parturition in mule deer relative to other species such as caribou or moose is likely related to the differing life history strategies exhibited by ungulate species. Because the offspring of moose and caribou species are more mobile after birth relative to mule deer offspring, maternal females can travel with recently born offspring and are not restricted to one area in the days following parturition [30]. However, follower offspring are still slow and vulnerable relative to adults, resulting in slower movements of maternal females overseeing their care [54, 55]. Thus, methods such as the PBM, IBM, and BCPA which incorporate movement rates and step-length metrics may be most successful at predicting parturition for ungulates with follower offspring. Conversely, because mule deer have hider offspring, they are spatially limited and must return often to the same general area to care for their young [30]. Because methods relying on movement rate are less effective at estimating parturition in mule deer than methods incorporating space use, this could indicate that mule deer do not slow their movement rates drastically relative to follower species, but still use less space on account of having to consistently return to hiding offspring.

We found little support for our hypothesis that methods using multiple metrics to detectparturition would perform better than those using one. While the API was one of the best performing methods and uses multiple metrics (velocity, turning angle and home range size), the rMCP method performed similarly well with just one. Although other studies have highlighted the value of using multiple metrics [32–34], home range size may be sufficient for detecting parturition of mule deer. The majority of the methods we tested showed poorer precision than suggested in previous studies [25–27]. This may be attributed to differing methods of validation; for the initial studies involving the IBM, PBM, BCPA, and rMCP, timing of parturition was validated using weekly aerial surveys [25, 27]. Aerial surveys allow for researchers to know the approximate time period that parturition occurred but are prone to bias and are often less precise than using VITs, which provide the exact date and time of parturition [17, 51]. Therefore, estimates of precision reported in prior studies that used aerial surveys may have been positively biased. Unlike the other movement-based methods, the Peterson method used birthdates that were validated using VITs [26]. However, the Peterson method was originally tested over a two-week time window, while we tested it over a seven-week time window (which encompassed the entire neonate birthing season at our study site). The API had greater precision than the Peterson method over this seven-week window, and performed better than most other methods when VITs were used as a method of parturition validation.

The API and rMCP may also be used to precisely estimate the mean date of parturition for ungulate populations. The API, rMCP,, and Peterson methods were able to correctly estimate the mean date of parturition for the study population, while the mean dates of parturition estimated by the BCPA, IBM, and PBM deviated from the true date of parturition by nine to twelve days. The mean date of parturition in ungulate populations may shift due to a variety of factors, including climate, maternal condition, and location [5, 15, 21]. An understanding of mean parturition dates is necessary for further examination of such factors.

Knowledge of both the rate and timing of parturition in ungulate populations is essential for exploring related ecological questions, such as how parturition is affected by maternal age, forage availability, and location [7, 13, 14, 56]. Knowledge of parturition in ungulate populations may also inform scientists of changes in population dynamics, as the timing of parturition may affect the survival, and therefore the recruitment, of neonates [57, 58]. The API or the rMCP method may be used to obtain information on rates and timing of parturition in mule deer, allowing for inferences to be made of neonatal survival, pregnancy rates, and impacts of environmental factors. Both methods were accurate (92%) at determining parturition status, and were precise within seven days of estimating the correct date of parturition (90%). Additionally, the API and rMCP correctly estimated the mean date of parturition of the study population. Therefore, these methods may be used to answer a variety of questions related to mule deer parturition without the time and effort normally required to do so.

### Supplementary Information

**Additional file1**: An additional file shows examples of nonparturient and parturient mule deer identified by the API, rMCP, IBM, PBM, BCPA, and Peterson methods.

## Data Availability

R code for the API, R code for other methods, and an example dataset are available on Github at https://github.com/TabHughes/Estimating-Parturition-of-Mule-Deer. Additional datasets used to support this study are available from the corresponding author upon request.

## Abbreviations

API: Analysis of parturition indicators
BCPA: Behavioral change point analysis
IBM: Individual-based method
PBM: Population-based method
rMCP: Rolling minimum convex polygons
VIT: Vaginal implant transmitter
