# Supplementary material for: Evaluating movement-based methods for estimating the frequency and timing of parturition in mule deer
Source: Mov Ecol. 2024 Jan 19;12:6. doi: 10.1186/s40462-024-00450-4 (PMC10799437; doi:10.1186/s40462-024-00450-4)
Supplement: Supplementary file 1 — Additional file1: An additional file shows examples of nonparturient and parturient mule deer identified by the API, rMCP, IBM, PBM, BCPA, and Peterson methods. [file 40462_2024_450_MOESM1_ESM.docx]

**Supplementary Information: Evaluating movement-based methods for estimating the frequency and timing of parturition in mule deer**

Tabitha A. Hughes^1*^, Randy T. Larsen^1^, Kent R. Hersey^2^, Madelon van de Kerk^3^ & Brock R. McMillan^1^

^1^Department of Plant and Wildlife Sciences, Brigham Young University, Provo, UT 84602

^2^Utah Division of Wildlife Resources, Salt Lake City, UT 84116

^3^ School of Environment and Sustainability, Western Colorado University, Gunnison, CO 81231


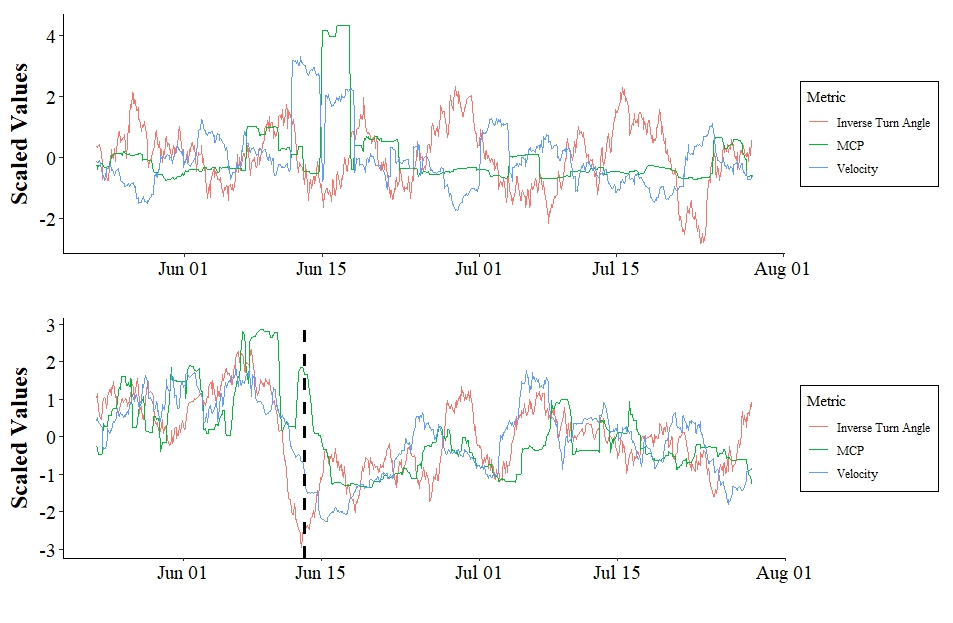


**Figure S1**. Example of a parturition date of a pregnant mule deer (bottom) identified by the API method, compared to deer identified as nonparturient (top). A parturition event is indicated by the dashed vertical line. Turning angle, velocity, and home range size are calculated over 3 day rolling intervals, with parturition identified once specified movement thresholds are met.


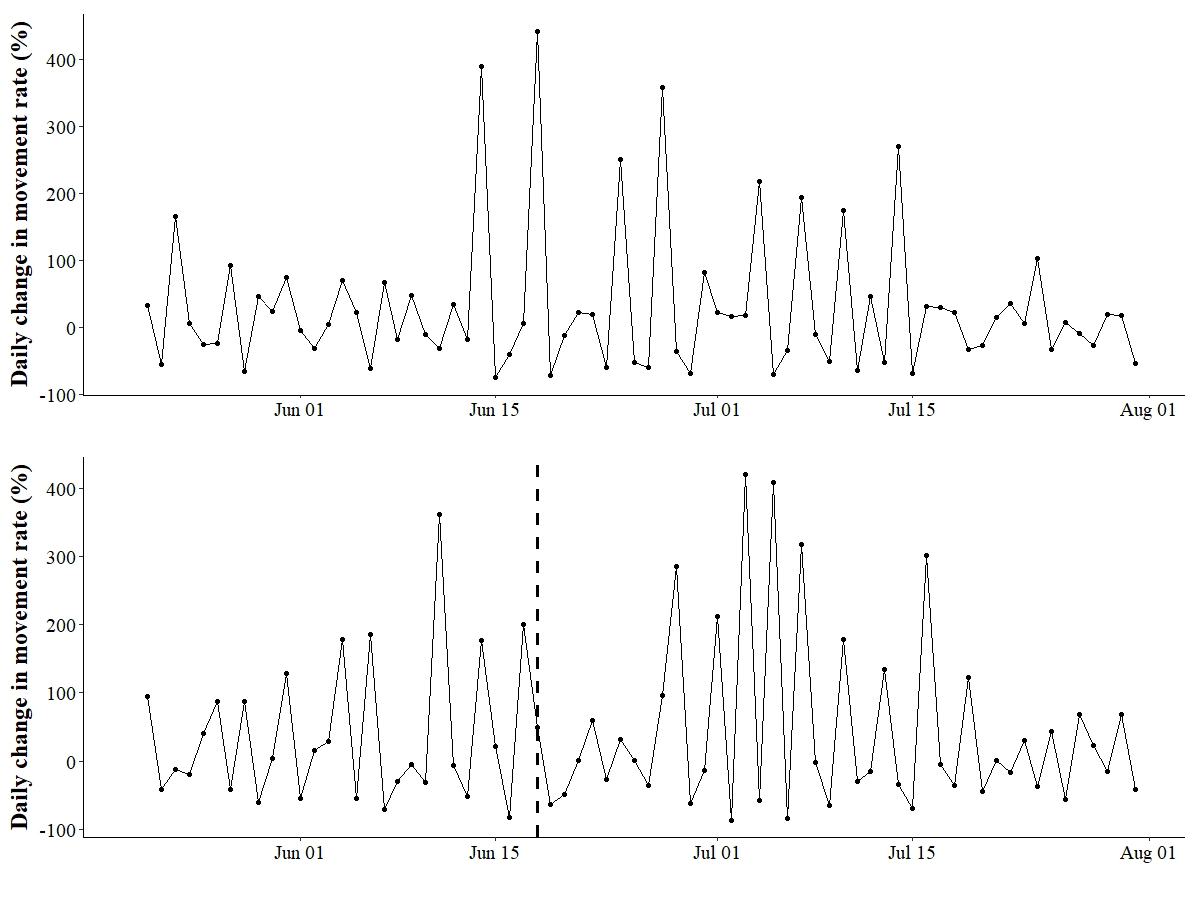


**Figure S2**. Example of a parturition date of a pregnant mule deer (bottom) identified by the Peterson method, compared to a deer identified as nonparturient (top). A parturition event is indicated by the dashed vertical line. Points indicate changes in daily movement rate, and parturition is identified once rates fall below a specified threshold and remain at this low level for at least three days.


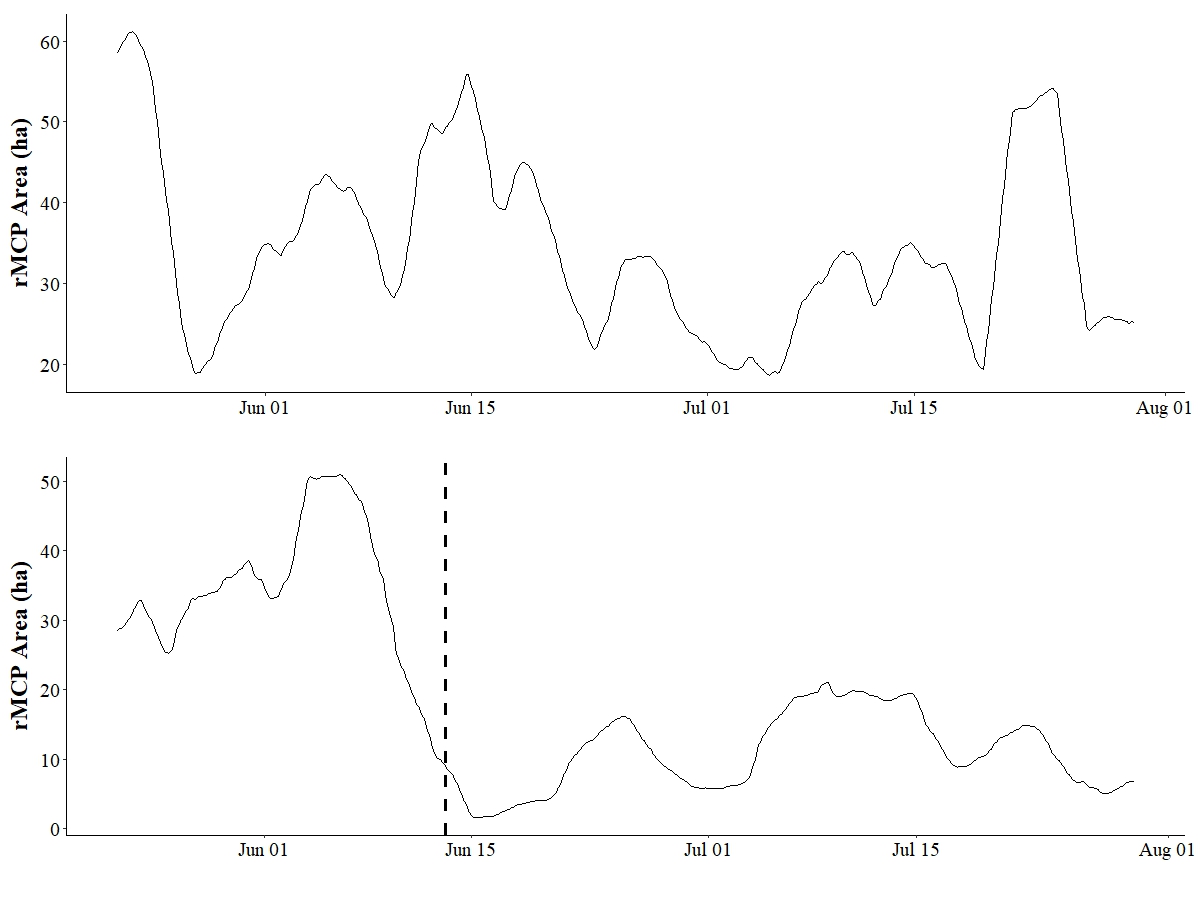


**Figure S3**. Example of a parturition date of a pregnant mule deer (bottom) identified by the rMCP method, compared to a deer identified as nonparturient (top). A parturition event is indicated by the dashed vertical line. Two-day rolling home range size averaged over 120 hour periods are projected in each figure. Parturition is identified once average home range size falls below a specified threshold.


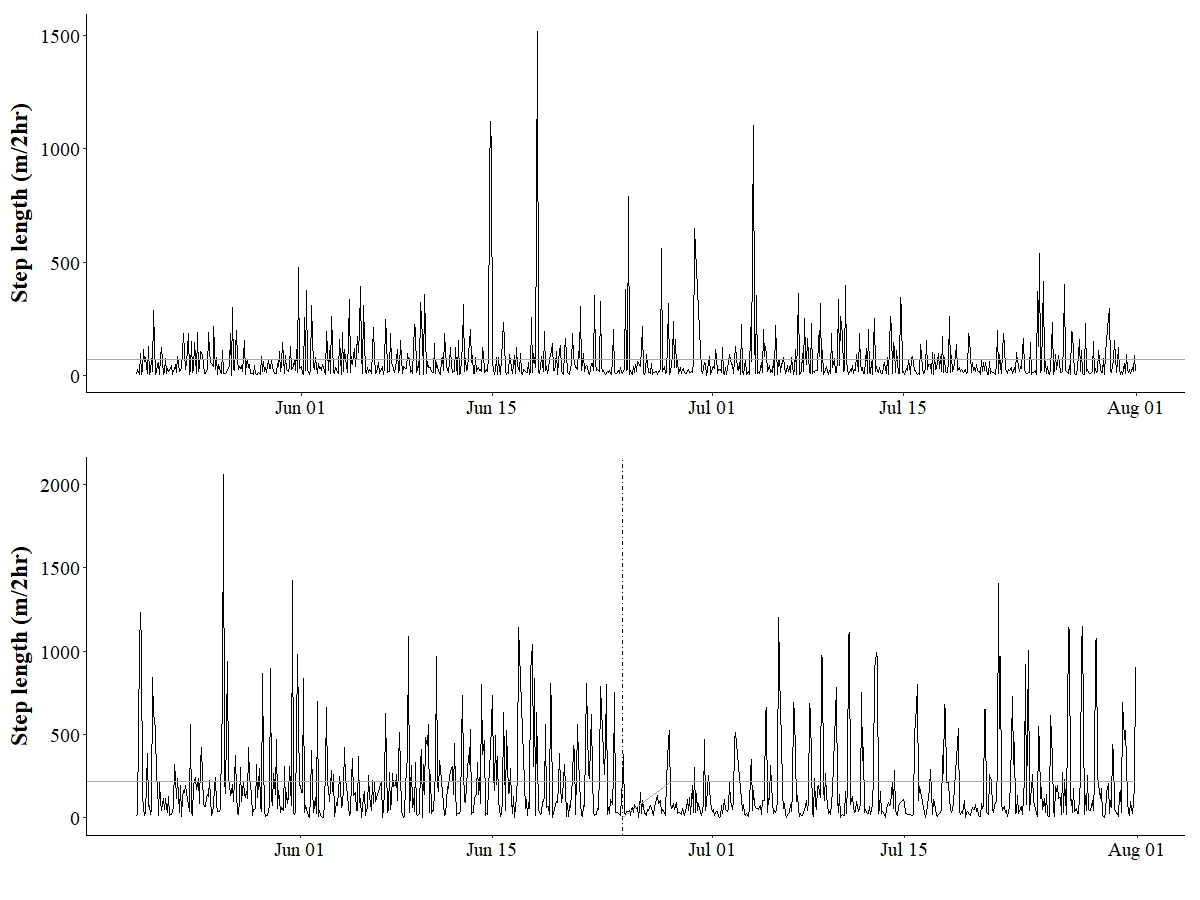


**Figure S4**. Example of a parturition date of a pregnant mule deer (bottom) identified by the IBM method, compared to a deer identified as nonparturient (top). A parturition event is indicated by the dashed vertical line. Step-length is used to predict parturition and is identified once movement rate falls below a specified threshold, accompanied by a slow increase to pre-parturient movement levels.


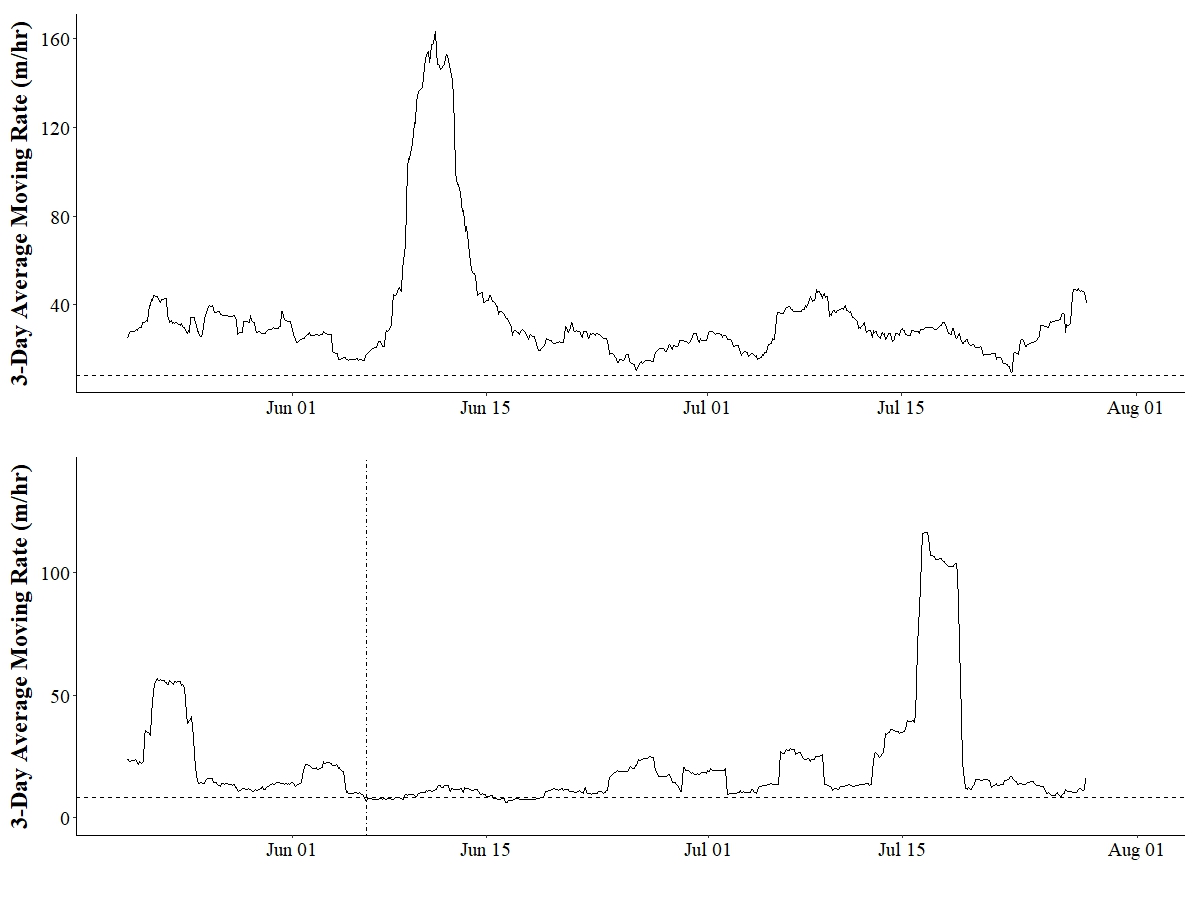
**Figure S5**. Example of a parturition date of a pregnant mule deer (bottom) identified by the PBM method, compared to a deer identified as nonparturient (top). A parturition event is indicated by the dashed vertical line. The movement threshold (indicated by the dashed horizontal line) is determined using post-parturient step-length data from a training population with known parturition dates. Parturition is identified once movement rates fall below this specified threshold.


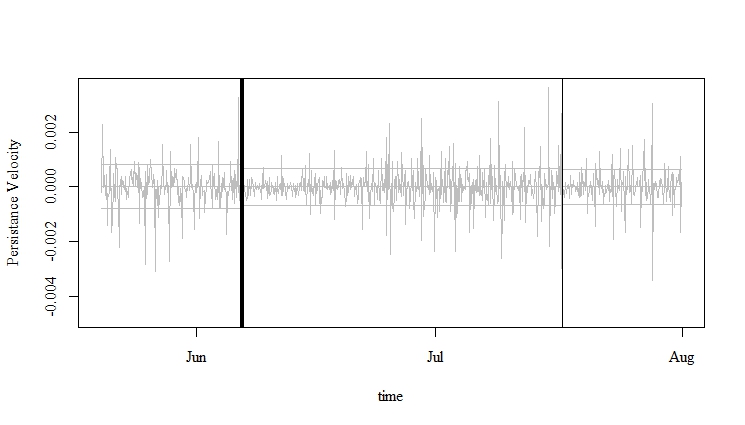

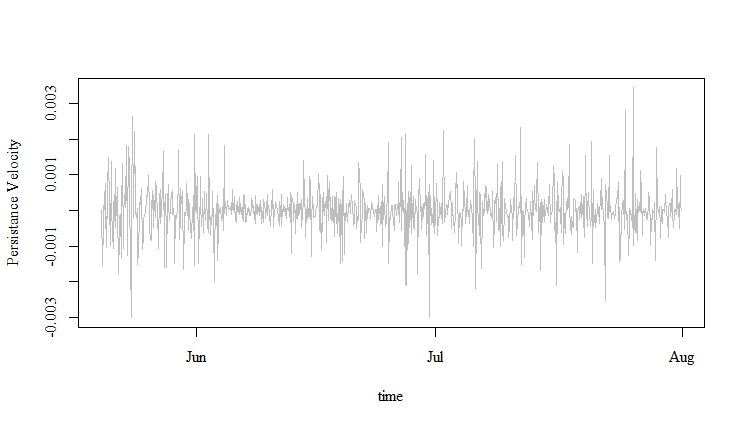


**Figure S6**. Example of a parturition date of a pregnant mule deer (bottom) identified by the BCPA method, compared to a deer identified as nonparturient (top). A parturition event is indicated by the solid vertical line. Persistence velocity is used to identify changepoints associated with changes in behavior indicative of parturition.
